# Ground state energy of a dilute Bose gas with three-body hard-core interactions

**DOI:** 10.1007/s11005-026-02067-7

**Published:** 2026-03-27

**Authors:** Lukas Junge, François L. A. Visconti

**Affiliations:** 1 Department of Mathematics, Copenhagen University, Lyngbyvej 2, 2100 Copenhagen, Denmark; 2 Department of Mathematics, LMU Munich, Theresienstrasse 39, 80333 Munich, Germany

**Keywords:** Dilute Bose gas, Three-body interaction, Hard core potential, BEC, Ground state energy

## Abstract

We consider a gas of bosons interacting through a three-body hard-core potential in the thermodynamic limit. We derive an upper bound on the ground state energy of the system at the leading order using a Jastrow factor. Our result matches the lower bound proven by Nam–Ricaud–Triay (J Math Phys 63:071903, 2022) and therefore resolves the leading order. Moreover, a straightforward adaptation of our proof can be used for systems interacting via combined two-body and three-body interactions to generalise Theorem 1.2 from (Ann. Henri Poincaré, 2026) to hard-core potentials.

## Introduction

A system of *N* bosons trapped in a box $\Lambda _L :=\left[ 0,L\right] ^3$ interacting via three-body interactions can be described by the Hamiltonian operator

1 $$\begin{aligned} H_{N,L} = \sum _{i=1}^N-\Delta _i + \sum _{1\le i<j<k\le N}w(x_i-x_j,x_i-x_k) \end{aligned}$$

acting on the Hilbert space $L_s ^2(\Lambda _L^N)$ - the subspace of $L^2(\Lambda _L^N)$ consisting of functions that are symmetric with respect to permutations of the *N* particles - and where $-\Delta$ denotes the Laplacian with Neumann boundary conditions on $\Lambda _L$ ($\nabla f \cdot \vec {n} = 0 on \partial \Lambda _L$). Such systems have received a lot of attention in recent years and have been the subject of many mathematical works [1, 5–7, 12, 13, 18–20, 22, 24, 25].

In [16], Nam–Ricaud–Triay proved that for a nonnegative, compactly supported potential $w\in L^\infty (\mathbb {R}^6)$, the Hamiltonian (1) satisfies

2 $$\begin{aligned} \quad \lim _{\begin{array}{c} N,L\rightarrow \infty \\ N/L^3\rightarrow \rho \end{array}}\dfrac{\inf \sigma (H_{N,L})}{N} = \dfrac{1}{6}\rho ^2b_{\mathcal {M}}(w)(1 + O((\rho b_\mathcal {M}(w)^{3/4})^\nu )) \end{aligned}$$

in the dilute regime $\rho b_\mathcal {M}(w)^{3/4} \rightarrow 0$, for some constant $\nu > 0$. Here, $\rho$ denotes the density of the system and $b_\mathcal {M}(w)$ is the scattering energy associated to *w*, which characterises the energy of three-particle collisions and is defined in (11). This result was then improved in [23], where it was shown that (2) holds for $w\ge 0$ compactly supported and satisfying

$$\begin{aligned} \Vert w\Vert _{L^2L^1} :=\Big (\int _{\mathbb {R}^3}\Vert w(x,\cdot )\Vert _{L^1(\mathbb {R}^3)}^2\operatorname {d}\!{}x\Big )^{1/2} < \infty . \end{aligned}$$

It was also shown in [23] that (2) holds with an error in *o*(1) for *w* of class $L^1$. The goal of this paper is to prove that (2) remains valid for particles interacting with a hard-core potential.

We consider a gas of *N* bosons with three-body hard-core interactions in $\Lambda _L = [0,L]^3$. We study the ground state energy

3 $$\begin{aligned} E_{N,L} = \inf \dfrac{\left<\Psi ,\sum _{i=1}^N-\Delta _i\Psi \right>}{\Vert \Psi \Vert ^2}, \end{aligned}$$

with the infimum taken over all $\Psi \in L_s ^2(\Lambda _L^N)$ satisfying the three-body hard-core condition $\Psi (x_1,\dots ,x_N) = 0$ if there exist $i,j,k\in \{1,\dots ,N\}$, $i\ne j\ne k\ne i$ with $|(x_i-x_j,x_i-x_k,x_j-x_k)|/\sqrt{3} \le \mathfrak {a}$.^1^ Here, $|\cdot |$ is the Euclidean norm in $\mathbb {R}^9$ and $\sum _{i=1}^N-\Delta _i$ is to be understood in the quadratic form sense in $H^1_s (\Lambda _L^N)$. The three-body scattering energy associated to the hard-core potential

$$\begin{aligned} w_hc (x-y,x-z) = \left\{ \begin{array}{ll} +\infty &  if |(x-y,x-z,y-z)|/\sqrt{3} \le \mathfrak {a}, \\ 0 &  otherwise \end{array} \right. \end{aligned}$$

is given by

4 $$\begin{aligned} b_\mathcal {M}(w_hc ) = \dfrac{64}{3\sqrt{3}}\pi ^2\mathfrak {a}^4. \end{aligned}$$

This is shown in the proof of Lemma 2.

Here is our main result.

### Theorem 1

There exist a constant $C > 0$ (independent of $\mathfrak {a}$ and $\rho$) such that

5 $$\begin{aligned} \lim _{\begin{array}{c} N,L\rightarrow \infty \\ N/L^3\rightarrow \rho \end{array}}\dfrac{E_{N,L}}{N} = \dfrac{32}{9\sqrt{3}}\pi ^2\rho ^2\mathfrak {a}^4\left( 1 + C\left( \rho \mathfrak {a}^3\right) ^{4/7}\right) \end{aligned}$$

for all $\rho \mathfrak {a}^3$ small enough.

In the present paper, we prove an upper bound on (5), which matches the lower bound proven in [16]. Here are some remarks on the result:

1. The strategy of the proof of Theorem 1 can be used to generalise (2) to *w* of class $L^1$ with an error uniform in *w* assuming that $R_0/b_\mathcal {M}(w)^{1/4}$ remains bounded, where $R_0$ is the range of *w*.
2. In [15, Conjecture 8], a heuristic approach was used to predict that the ground state energy of a system described by the Hamiltonian (1) should satisfy
  6 $$\begin{aligned} \quad \lim _{\begin{array}{c} N,L\rightarrow \infty \\ N/L^3\rightarrow \rho \end{array}}\dfrac{\inf \sigma (H_{N,L})}{N} = \dfrac{1}{6}\rho ^2b_{\mathcal {M}}(w)(1 + C(w)\rho + o(\rho )) \end{aligned}$$
  in the low density regime $\rho \rightarrow 0$, for some constant *C*(*w*) depending only on *w*. The error yielded by the proof of Theorem 1 is of order $(\rho \mathfrak {a}^3)^{4/7} \gg \rho \mathfrak {a}^3$, meaning that it does not capture the error at the correct order. The same problem arises in the two-body case when only using cancellations between the numerator and the denominator similar to the ones used in (22)–(24) (see for example [3]). To extract an error at the correct order in the two-body case one needs to push the analysis much further and identify additional cancellations, as was done in [2].
3. A straightforward adaptation of the proof of Theorem 1 can be used to derive a correct upper bound at the first order of the ground state energy of a system interacting via combined two-body and three-body interactions. More specifically, the ground state energy $E_{N,L}'$ of a system of *N* bosons located in $\Lambda _L$ interacting via a two-body hard-core potential of radius $\mathfrak {a}_2$ and a three-body hard-core potential of radius $\mathfrak {a}_3 > \mathfrak {a}_2$ is such that
  $$\begin{aligned} \qquad \quad \lim _{\begin{array}{c} N,L\rightarrow \infty \\ N/L^3\rightarrow \rho \end{array}}\dfrac{E_{N,L}'}{N} = \Big (4\pi \rho \mathfrak {a}_2 + \dfrac{32}{9\sqrt{3}}\pi ^2\rho ^2\mathfrak {a}_3 ^4\Big ) \left( 1 + C\left( \rho \mathfrak {a}_2 ^3\right) ^{\nu _1} + C\left( \rho \mathfrak {a}_3 ^3\right) ^{\nu _2}\right) \end{aligned}$$
  for all $\rho \mathfrak {a}_2^3$ and $\rho \mathfrak {a}_3^3$ small enough and for some $\nu _1,\nu _2 >0$ (independent of $\mathfrak {a}_2, \mathfrak {a}_3$ and $\rho$). This generalises [23, Theorem 1.2] to hard-core potentials. The lower bound proven in [23] easily generalises to the hard-core case, whereas the proof of the upper bound, which relies on second quantisation techniques, fails. Instead, one proves the upper bound using a trial state of the form
  $$\begin{aligned} \qquad \Psi (x_1,\dots ,x_N) = \prod _{1\le i<j\le N}\widetilde{f}_{\ell _1}(x_i - x_j)\prod _{1\le i<j<k\le N}f_{\ell _2}(x_i - x_j,x_i - x_k), \end{aligned}$$
  where $\widetilde{f}_{\ell _1}$ describes the two-body correlations up to a distance $\ell _1$ and $f_{\ell _2}$ describes the three-body correlations up to a distance $\ell _2$. Note that this trial state is simply the product of the trial considered in the proof of Theorem 1 (see (18)) and the standard trial state used in the two-body case.

## Scattering properties of the three-body hard-core potential

Since we are considering a dilute gas, the correlation structure is encoded in the zero-energy scattering problem

7 $$\begin{aligned} (-\Delta _x -\Delta _y -\Delta _z)f(x-y,x-z) = 0 \end{aligned}$$

on $\mathbb {R}^9$, where *f* satisfies the conditions $f(x-y,x-z) = 0$ if $|(x-y,x-z,y-z)|/\sqrt{3} \le \mathfrak {a}$ and $f(\textbf{x}) \rightarrow 1$ as $|\textbf{x}| \rightarrow \infty$. Note that *f* satisfies the three-body symmetry properties

8 $$\begin{aligned} f(x,y) = f(y,x) \quad and \quad f(x - y,x - z) = f(y - x,y - z) = f(z - x, z - y), \end{aligned}$$

for all $$x,y,z\in \mathbb {R}^3$$.

By removing the centre of mass using the change of variables

9 $$\begin{aligned} r_1 = \dfrac{1}{3}(x + y + z), \quad r_2 = x-y, \quad and \quad r_3 = x-z, \end{aligned}$$

we find that the scattering problem (7) is equivalent to the modified zero-energy scattering problem

10 $$\begin{aligned} -2\Delta _\mathcal {M}f(r_2,r_3) = 0 \end{aligned}$$

on $\mathbb {R}^6$, with *f* satisfying the conditions $f(r_2,r_3) = 0$ if $\left| \mathcal {M}^{-1}(r_2,r_3)\right| \le \sqrt{2}\mathfrak {a}$ and $f(\textbf{x}) \rightarrow 1$ as $|\textbf{x}| \rightarrow \infty$. Here, we introduced the modified Laplacian

$$\begin{aligned} -\Delta _\mathcal {M} = -|\mathcal {M}\nabla _{\mathbb {R}^6}|^2 = -\operatorname {div}_{\mathbb {R}^6}(\mathcal {M}^2\nabla _{\mathbb {R}^6}), \end{aligned}$$

where the matrix $\mathcal {M}:\mathbb {R}^3\times \mathbb {R}^3 \rightarrow \mathbb {R}^3\times \mathbb {R}^3$ is given by

$$\begin{aligned} \mathcal {M} :=\left( \dfrac{1}{2} \begin{pmatrix} 2 &  1\\ 1 &  2 \end{pmatrix} \right) ^{1/2} = \dfrac{1}{2\sqrt{2}} \begin{pmatrix} \sqrt{3} + 1 &  \sqrt{3} - 1\\ \sqrt{3} - 1 &  \sqrt{3} + 1 \end{pmatrix}, \end{aligned}$$

with inverse

$$\begin{aligned} \mathcal {M}^{-1} = \left( \dfrac{2}{3} \begin{pmatrix} 2 &  -1\\ -1 &  2 \end{pmatrix} \right) ^{1/2} = \dfrac{1}{\sqrt{6}} \begin{pmatrix} 1 + \sqrt{3} &  1 - \sqrt{3}\\ 1 - \sqrt{3} &  1 + \sqrt{3} \end{pmatrix}. \end{aligned}$$

Note that $\det \mathcal {M} = \sqrt{3}/2$. Define the scattering energy $b_\mathcal {M}(w)$ of some three-body potential *w* by

11 $$\begin{aligned} b_\mathcal {M}(w) = \inf _{g\in D^1(\mathbb {R}^6)}\int _{\mathbb {R}^6}\operatorname {d}\!{}\textbf{x}\left( 2\vert \mathcal {M}\nabla g(\textbf{x})\vert ^2 + w(\textbf{x})\vert 1 - g(\textbf{x})\vert ^2\right) , \end{aligned}$$

where $D^1(\mathbb {R}^6)$ denotes the space of functions $g:\mathbb {R}^6 \rightarrow \mathbb {C}$ in $L^1_{loc }(\mathbb {R}^6)$ vanishing at infinity and such that $\vert \nabla g\vert \in L^2(\mathbb {R}^6)$ (see [14, Section 8.3]). We refer to [17] for a more in-depth discussion on the matter.

Let *f* denote a solution to (10) and define $\tilde{f} = f(\mathcal {M}\cdot )$. Then, $\tilde{f}$ solves

$$\begin{aligned} -\Delta \tilde{f} = 0 \end{aligned}$$

on $\mathbb {R}^6$, with the conditions $\tilde{f}(\textbf{x}) = 0$ for $|\textbf{x}| \le \sqrt{2}\mathfrak {a}$ and $\tilde{f}(\textbf{x}) \rightarrow 1$ as $|\textbf{x}| \rightarrow \infty$. By rewriting the previous problem in hyperspherical coordinates we find that (10) has for unique solution

$$\begin{aligned} f(\textbf{x}) = \left\{ \begin{array}{ll} 1 - \dfrac{4\mathfrak {a}^4}{|\mathcal {M}^{-1}\textbf{x}|^4} \quad & if |\mathcal {M}^{-1}\textbf{x}| > \sqrt{2}\mathfrak {a} ,\\ 0 & otherwise . \end{array} \right. \end{aligned}$$

Define also

$$\begin{aligned} \omega (\textbf{x}) = 1 - f(\textbf{x}), \end{aligned}$$

for all $$\textbf{x}\in \mathbb {R}^6$$.

We shall need a truncated version of *f* with a cut-off. Let $\tilde{\chi }\in C^\infty (\mathbb {R}^6;[0,1])$ be a radial function satisfying $\tilde{\chi }(\textbf{x}) = 1$ if $|\textbf{x}|\le 1/2$ and $\tilde{\chi }(\textbf{x}) = 0$ if $|\textbf{x}| \ge 1$, and define $\chi = \tilde{\chi }(\mathcal {M}^{-1}\cdot )$. For all $\ell \in (\mathfrak {a},L)$, we define

12 $$\begin{aligned} \chi _\ell = \chi (\ell ^{-1}\cdot ), \quad \omega _\ell = \chi _\ell \omega , \quad and \quad f_\ell = 1 - \omega _\ell . \end{aligned}$$

Note that $\omega , f, \omega _\ell$ and $f_\ell$ satisfy the three-body symmetry (8). Moreover, they have the following properties:

### Lemma 2

Let $$\ell \in (\mathfrak {a},L)$$. Then,

13 $$\begin{aligned} |\nabla f_\ell (\textbf{x})| \le C\mathfrak {a}^4\dfrac{1\!\!1_{\left\{ C_1\mathfrak {a}\le |\textbf{x}|\le C_2\ell \right\} }}{|\textbf{x}|^5} \end{aligned}$$

and

14 $$\begin{aligned} 0 \le 1 - f_\ell ^2(\textbf{x}) \le C\mathfrak {a}^4\dfrac{1\!\!1_{\left\{ C_1\mathfrak {a}\le |\textbf{x}|\le C_2\ell \right\} }}{|\textbf{x}|^4}, \end{aligned}$$

for all $\textbf{x}\in \mathbb {R}^6$. Here, $C,C_1,C_2$ are universal positive constants such that $C_1 < C_2$. Moreover,

15 $$\begin{aligned} \int _{\mathbb {R}^6}\operatorname {d}\!{}\textbf{x}\left( |(\mathcal {M}\nabla _{\mathbb {R}^6}f_\ell )(\textbf{x})|^2\right) \le \dfrac{32}{3\sqrt{3}}\pi ^2\mathfrak {a}^4\Big (1 + C\Big (\dfrac{\mathfrak {a}}{\ell }\Big )^4\Big ) \end{aligned}$$

Furthermore, define $\tilde{\ell } = \sqrt{3/2}\ell$ and

16 $$\begin{aligned} g_\ell (x) = 1\!\!1(|x| \ge \tilde{\ell }). \end{aligned}$$

Then,

17 $$\begin{aligned} f_\ell (x_1,x_2) \ge \max (g_\ell (x_1),g_\ell (x_2)), \end{aligned}$$

for all $$x_1,x_2\in \mathbb {R}^3$$.

### Proof

Both (13) and (14) follow directly from the definition of $f_\ell$ and $|\nabla \chi _\ell (\textbf{x})| \le C\ell ^{-1}1\!\!1_{\left\{ \ell /2\le |\mathcal {M}^{-1}\textbf{x}|\le \ell \right\} }$ and $\sigma (\mathcal {M}^{-1}) = \left\{ \sqrt{2/3},\sqrt{2}\right\}$. To compute (15) we first expand

$$\begin{aligned} \int _{\mathbb {R}^6}\operatorname {d}\!{}\textbf{x}\Big (|(\mathcal {M}\nabla _{\mathbb {R}^6}f_\ell )(\textbf{x})|^2\Big )&= \int _{\mathbb {R}^6}\operatorname {d}\!{}\textbf{x}\Big (|(\mathcal {M}\nabla _{\mathbb {R}^6}\omega )(\textbf{x})|^2\chi _\ell (\textbf{x})^2\Big )\\&\phantom {=} + 2\int _{\mathbb {R}^6}\operatorname {d}\!{}\textbf{x}\Big ((\mathcal {M}\nabla _{\mathbb {R}^6}\omega )(\textbf{x})\cdot (\mathcal {M}\nabla _{\mathbb {R}^6} \chi _\ell )(\textbf{x})\omega _\ell (\textbf{x})\Big )\\&\phantom {=} + \int _{\mathbb {R}^6}\operatorname {d}\!{}\textbf{x}\Big (\omega (\textbf{x})^2|(\mathcal {M}\nabla _{\mathbb {R}^6}\chi _\ell )(\textbf{x})|^2\Big ). \end{aligned}$$

The only contribution of order $\mathfrak {a}^4$ comes from the first term. Indeed, using again $|\nabla \chi (\textbf{x})| \le C\ell ^{-1}1\!\!1_{\left\{ \ell /2\le |\mathcal {M}^{-1}\textbf{x}|\le \ell \right\} }$ and (13) we have

$$\begin{aligned} \int _{\mathbb {R}^6}\operatorname {d}\!{}\textbf{x}\Big (2(\mathcal {M}\nabla _{\mathbb {R}^6}\omega )(\textbf{x})\cdot (\mathcal {M}\nabla _{\mathbb {R}^6} \chi _\ell )(\textbf{x})\omega _\ell (\textbf{x}) + |\omega (\textbf{x})^2|(\mathcal {M}\nabla _{\mathbb {R}^6}\chi _\ell )(\textbf{x})|^2\Big )\\ \le C\mathfrak {a}^4\left( \dfrac{\mathfrak {a}}{\ell }\right) ^4. \end{aligned}$$

Moreover, by writing $\omega = \tilde{\omega }(\mathcal {M}^{-1}\cdot )$ with $\tilde{\omega }(\textbf{x}) = 4\mathfrak {a}^4/|\textbf{x}|^4$ if $\vert \textbf{x}\vert > \sqrt{2}\mathfrak {a}$ and $\tilde{\omega }(\textbf{x}) = 1$ otherwise, we get

$$\begin{aligned} \int _{\mathbb {R}^6}\operatorname {d}\!{}\textbf{x}\Big (|(\mathcal {M}\nabla _{\mathbb {R}^6}\omega )(\textbf{x})|^2\chi _\ell (\textbf{x})^2\Big )&= \int _{\mathbb {R}^6}\operatorname {d}\!{}\textbf{x}\Big (\left| (\nabla _{\mathbb {R}^6}\tilde{\omega })(\mathcal {M}^{-1}\textbf{x})\right| ^2\tilde{\chi }_\ell (\mathcal {M}^{-1}\textbf{x})^2\Big )\\&= \det \mathcal {M}\int _{\mathbb {R}^6}\operatorname {d}\!{}\textbf{y}\Big (\left| (\nabla _{\mathbb {R}^6}\tilde{\omega })(\textbf{y})\right| ^2\tilde{\chi }_\ell (\textbf{y})^2\Big )\\&\le \dfrac{32}{3\sqrt{3}}\pi ^2\mathfrak {a}^4. \end{aligned}$$

In the second equality we used the change of variables $\textbf{y} = \mathcal {M}^{-1}\textbf{x}$. In the last inequality we used $\nabla _{\mathbb {R}^6}\tilde{\omega } = 0$ on $B(0,\sqrt{2}\mathfrak {a})$ and $\tilde{\chi }_\ell (\textbf{x}) \le 1\!\!1_{\left\{ |\textbf{x}| \le \ell \right\} }$ and $\nabla _\textbf{y}(1/|\textbf{y}|^4) = -4\textbf{y}/|\textbf{y}|^6$ and $\det \mathcal {M} = \sqrt{3}/2$ and that the surface of the 5-dimensional sphere in $\mathbb {R}^6$ is given by $|\mathbb {S}^5| = 8\pi ^2/3$. This proves (15). Notice also that the same computation gives (4).

Finally, notice that $f_\ell (x_1,x_2) = 1$ when $|\mathcal {M}^{-1}(x_1,x_2)|^{-1}\ge \ell$, which is true whenever $|x_1| \ge \tilde{\ell }$ or $|x_2| \ge \tilde{\ell }$. This immediately implies (17) and concludes the proof of Lemma 2. $\square$

## Proof of the upper bound

To get an upper bound on (3), we need to evaluate the energy on an appropriate trial state. To do so, we add correlations among particles to the uncorrelated state $\tilde{\Psi }_{N,L} \equiv 1$. Since correlations are produced mainly by three-body scattering events, we consider the trial state

18 $$\begin{aligned} \Psi _{N,L}(x_1,\dots ,x_N) = \prod _{1\le i<j<k\le N}f_\ell (x_i-x_j,x_i-x_k), \end{aligned}$$

where $\ell$ is a parameter satisfying $\mathfrak {a} \ll \ell \ll L$ that will be fixed later; the state $\Psi _{N,L}$ clearly satisfies the three-body hard-core condition imposed in (3). The function $f_\ell$ defined in (12) describes the three-body correlations up to a distance $\ell$. Trial states of this form have been first used in [4, 9, 11] in the two-body case and are usually referred to as Jastrow factors. Note that though Dyson [10] worked with a nonsymmetric trial state describing only nearest-neighbour correlations, trial states similar to (18) can be used to derive the leading order of a Bose gas with two-body interactions, as shown in [3]. For readability’s sake, we from now on write

$$\begin{aligned} f_{ijk} = f_\ell (x_i-x_j,x_i-x_k) \end{aligned}$$

and

19 $$\begin{aligned} \nabla _if_{ijk} = \nabla _{x_i}f_\ell (x_i-x_j,x_i-x_k) \end{aligned}$$

for all $$i,j,k\in \{1,\dots ,N\}$$.

To compute the energy of the trial state (18), we first notice that

$$\begin{aligned} \nabla _{x_1}\Psi _{N,L}(x_1,\dots ,x_N) = \sum _{2\le p<q \le N}\dfrac{\nabla _{1}f_{1pq}}{f_{1pq}}\prod _{1\le i<j<k\le N}f_{ijk}, \end{aligned}$$

which when combined with the three-body symmetry (8) implies

$$\begin{aligned}\begin{array}{c} \dfrac{\left<\Psi _{N,L},\sum _{i=1}^N-\Delta _i\Psi _{N,L}\right>}{\Vert \Psi _{N,L}\Vert ^2} = N\dfrac{\left<\nabla _1\Psi _{N,L},\nabla _1\Psi _{N,L}\right>}{\Vert \Psi _{N,L}\Vert ^2} \\ \begin{aligned} & = \dfrac{N(N-1)(N-2)}{3}\dfrac{\int \operatorname {d}\!{}\textbf{x}_N\left( \frac{|\mathcal {M}\nabla f_{123}|^2}{f_{123}^2}\prod _{1\le i<j<k\le N}f_{ijk}^2\right) }{\int \operatorname {d}\!{}\textbf{x}_N\left( \prod _{1\le i<j<k\le N}f_{ijk}^2\right) } \\ & \phantom {=} + N(N-1)(N-2)(N-3)\dfrac{\int \operatorname {d}\!{}\textbf{x}_N\left( \frac{\nabla _1f_{123}}{f_{123}}\cdot \frac{\nabla _1f_{124}}{f_{124}}\prod _{1\le i<j<k\le N}f_{ijk}^2\right) }{\int \operatorname {d}\!{}\textbf{x}_N\left( \prod _{1\le i<j<k\le N}f_{ijk}^2\right) } \\ & \phantom {=} + \dfrac{N(N-1)(N-2)(N-3)(N-4)}{4}\dfrac{\int \operatorname {d}\!{}\textbf{x}_N\left( \frac{\nabla _1f_{123}}{f_{123}}\cdot \frac{\nabla _1f_{145}}{f_{145}}\prod _{1\le i<j<k\le N}f_{ijk}^2\right) }{\int \operatorname {d}\!{}\textbf{x}_N\left( \prod _{1\le i<j<k\le N}f_{ijk}^2\right) } \\ & {=}{:}\mathcal {I}_1 + \mathcal {I}_2 + \mathcal {I}_3. \end{aligned} \end{array}\end{aligned}$$

In the second equality we used

$$\begin{aligned}\begin{array}{c} \left| \nabla _{x_1}f_\ell (x_1 - x_2,x_1 - x_3)\right| ^2 + \left| \nabla _{x_2}f_\ell (x_1 - x_2,x_1 - x_3)\right| ^2 \\ + \left| \nabla _{x_3}f_\ell (x_1 - x_2,x_1 - x_3)\right| ^2 = 2\left| (\mathcal {M}\nabla _{\mathbb {R}^6} f_\ell )(x_1 - x_2,x_1 - x_3)\right| ^2. \end{array}\end{aligned}$$

Let us now bound each term one by one. Thanks to (17), we have

20 $$\begin{aligned} \prod _{3\le j<k\le N}f_\ell (x_i-x_j,x_i-x_k)^2 \ge \prod _{j=3}^Ng_\ell (x_i - x_j), \end{aligned}$$

for $i\in \{1,2\}$. Hence, by defining $u_\ell = 1 - f_\ell ^2$ and $v_\ell = 1 - g_\ell$ we have the estimate

21 $$\begin{aligned} 1 - \sum _{j=3}^Nv_{1j} - \sum _{j=3}^Nv_{2j} - \sum _{k = 3}^Nu_{12k} \le \prod _{3\le j<k\le N}f_{1jk}^2f_{2jk}^2\prod _{k=3}^Nf_{12k}^2 \le 1, \end{aligned}$$

where we used the short-hand notations $v_{ij} = v_\ell (x_i - x_j)$ and $u_{ijk} = u_\ell (x_i-x_j,x_i-x_k)$. Notice that, thanks to (20), the left-hand side of (21) contains solely single sums, and no double sums as it would have if we did not use this bound; this plays an important role in the estimates below. The bound (21) allows us to decouple the variables $x_1$ and $x_2$ in the numerator and in the denominator of $\mathcal {I}_1$; with (14) and (15) we obtain

22 $$\begin{aligned} \mathcal {I}_1&\le \dfrac{N^3}{3}\dfrac{\int _{\mathbb {R}^6}\operatorname {d}\!{}\textbf{x}\left( |(\mathcal {M}\nabla _{\mathbb {R}^6}f_\ell )(\textbf{x})|^2\right) }{L^6 - CL^3N\int \operatorname {d}\!{}x(v_\ell (x)) - CN\int \operatorname {d}\!{}\textbf{x}(u_\ell (\textbf{x}))} \nonumber \\&\le \dfrac{32}{9\sqrt{3}}\pi ^2\dfrac{N\rho ^2\mathfrak {a}^4(1 + C(\mathfrak {a}/\ell )^4)}{1 - C\rho \ell ^3 - C\rho \ell ^2\mathfrak {a}^4/L^3}\nonumber \\&\le \dfrac{32}{9\sqrt{3}}\pi ^2N\rho ^2\mathfrak {a}^4\Big (1 + C\Big (\dfrac{\mathfrak {a}}{\ell }\Big )^4 + C\rho \ell ^3\Big ), \end{aligned}$$

under the assumption that $\rho \ell ^3 \ll 1$ and $\mathfrak {a}\ll \ell \ll L$. In the last inequality we used $\ell ^2\mathfrak {a}^4/L^3 \le \ell ^3$. To bound $\mathcal {I}_2$ we similarly decouple the variables $x_2, x_3$ and $x_4$. Using again (13) and (14) we can bound

23 $$\begin{aligned} \mathcal {I}_2&\le CN^4\dfrac{\int \operatorname {d}\!{}x\operatorname {d}\!{}y\operatorname {d}\!{}z(|\nabla f_\ell (x,y)|\cdot |\nabla f_\ell (x,z)|)}{L^9 - CNL^6\int \operatorname {d}\!{}x(v_\ell (x)) - CNL^3\int \operatorname {d}\!{}\textbf{x}(u_\ell (\textbf{x}))}\nonumber \\&\le CN\rho ^2\mathfrak {a}^4\left[ \rho \mathfrak {a}^4\ell \right] \left( 1 + C\rho \ell ^3\right) \nonumber \\&\le CN\rho ^2\mathfrak {a}^4\left( \rho \ell ^3\right) , \end{aligned}$$

when $\rho \ell ^3 \ll 1$ and $\mathfrak {a}\ll \ell \ll L$. Analogously, we bound $\mathcal {I}_3$ by decoupling the variables $x_1,x_2$ and $x_4$. Namely, using once more (13) and (14) we get

24 $$\begin{aligned} \mathcal {I}_3&\le CN^5\dfrac{\left( \int \operatorname {d}\!{}\textbf{x}|\nabla f_\ell (\textbf{x})|\right) ^2}{L^{12} - CNL^9\int \operatorname {d}\!{}x(v_\ell (x)) - CNL^6\int \operatorname {d}\!{}\textbf{x}(u_\ell (\textbf{x}))} \nonumber \\&\le CN\rho ^2\mathfrak {a}^4\left[ \rho ^2\mathfrak {a}^4\ell ^2\right] \left( 1 + C\rho \ell ^3\right) \nonumber \\&\le CN\rho ^2\mathfrak {a}^4\left( \rho \ell ^3\right) \end{aligned}$$

again under the condition that $\rho \ell ^3 \ll 1$ and $\mathfrak {a}\ll \ell \ll L$. From (22)–(24) we conclude that

$$\begin{aligned} E_{N,L} \le \dfrac{32}{9\sqrt{3}}\pi ^2N\rho ^2\mathfrak {a}^4\Big (1 + C\Big (\dfrac{\mathfrak {a}}{\ell }\Big )^4 + C\rho \ell ^3\Big ). \end{aligned}$$

Taking $\ell = \mathfrak {a}\left( \rho \mathfrak {a}^3\right) ^{-1/7}$ finishes the proof of Theorem 1.

## Data Availability

Data sharing not applicable to this article as no datasets were generated or analysed during the current study.

## Footnotes

1 Though there is no canonical choice for the three-body hard-core potential, the present choice is motivated by the physics literature (see e.g. [8, 21]).
